# Effects of ivabradine and atenolol on heart rate and heart rate variability in healthy cats over a 24 h period: A pilot study

**DOI:** 10.1002/vro2.28

**Published:** 2022-02-09

**Authors:** Mizuki Ogawa, Ayano Kawamura, Ryota Akabane, Atsushi Sakatani, Hirosumi Miyakawa, Huai‐Hsun Hsu, Yuichi Miyagawa, Naoyuki Takemura

**Affiliations:** ^1^ Laboratory of Veterinary Internal Medicine II School of Veterinary Medicine Nippon Veterinary and Life Science University 1‐7‐1 Kyonan‐cho, Musashino‐shi Tokyo Japan; ^2^ Sakatani Animal Hospital 2070‐1 Arino‐cho, Kobe‐shi Hyogo Japan

## Abstract

**Background:**

Ivabradine is used to treat tachycardia; unlike atenolol, it does not affect blood pressure or myocardial contractility. This study compared the impact of ivabradine and atenolol on heart rate (HR) and HR variability (HRV) during a 24 h period, feeding and sleeping times, via a Holter electrocardiogram in healthy cats. We hypothesised that ivabradine and atenolol would lower the HRs equally well, even at times of excitement and rest, such as during feeding and sleep; that ivabradine, unlike atenolol, would have an effect on HRV.

**Methods:**

Five clinically healthy cats were used in the prospective blinded crossover study receiving 3 days of ivabradine (0.30 mg/kg per os twice daily) followed by atenolol (6.25 mg/cat per os twice daily, range 1.3–2.0 mg/kg) or receiving atenolol followed by ivabradine. A placebo period was initiated before the start of the crossover test, data obtained during that period were used as a baseline (BL). Evaluation parameters included HR and HRV, for the whole 24 h period and for feeding and sleeping times, comparing the effect of ivabradine and atenolol with BL.

**Results:**

The HR for the whole 24 h, feeding and sleeping times, were significantly lower with ivabradine and atenolol, compared to BL (*p* < 0.05). The HRV for the whole 24 h and sleeping time were significantly higher after ivabradine compared with BL and after atenolol.

**Conclusions:**

In healthy cats, ivabradine and atenolol significantly reduced the HR regardless of excitement and rest; their effects were comparable. Ivabradine significantly increased HRV in comparison to BL whereas atenolol did not.

## INTRODUCTION

Cardiomyopathies are a heterogeneous group of myocardial disorders of mostly unknown aetiology and they occur commonly in cats.^1^ In particular, hypertrophic cardiomyopathy (HCM) may be associated with dynamic left ventricular outflow tract obstruction (DLVOTO)^2^ although DLVOTO is not considered a prognostic indicator in cats.^3^, ^4^ Treatment of subclinical/asymptomatic cardiomyopathy in cats with HCM is controversial while treatment of severe DLVOTO may be considered.^1^, ^5^, ^6^, ^7^ Tachycardia increases myocardial ischaemia and contractility by shortening diastole and limiting diastolic coronary blood flow; myocardial ischaemia may in turn lead to cardiac fibrosis and increased left ventricular end‐diastolic pressure.^8^ Atenolol, a beta‐blocker, may be administered to cats to reduce the gradient of DLVOTO and the heart rate (HR).^2^ However, because atenolol can cause hypotension and reduced myocardial contractility, it can exacerbate the clinical condition of cats with congestive heart failure.^9^, ^10^

Ivabradine is specific to the pacemaker funny current (*I*_f_) channel inhibitor; it is used to treat ischaemic heart disease in human beings.^11^ Ivabradine can decrease the HR and myocardial oxygen consumption with no effects on blood pressure or myocardial contractility by selectively inhibiting the *I*_f_ channel in the sinoatrial node.^12^ Previous studies have reported that ivabradine and atenolol improve the DLVOTO in cats.^13^ A previous study in which ivabradine and atenolol were administered to healthy cats reported significant decreases in the HR compared to baseline (BL); their effects were similar, while treatment with ivabradine resulted in more favourable echocardiographic indices.^14^

In anaesthetized cats, the combined use of ivabradine, or esmolol, with dobutamine reduced HR only when ivabradine was used.^15^ It has also been reported that ivabradine significantly reduced HR in conscious cats during excitement.^16^ However, these studies only monitored HR instantaneously and no study, to the best of the authors’ knowledge, has monitored these outcomes for a 24 h period. A diurnal variation has been known to exist in the HR of cats. A study on cats reported that the mean HR increased from mid‐afternoon to approximately 9:00 PM and was lowest just after midnight.^17^ Moreover, another study reported that the population of cats showed the main peaks of activity in the morning, especially before sunrise and during feeding times.^18^ These findings suggest that the HR‐reducing effects of ivabradine and atenolol may be affected by feeding and sleeping. Accordingly, the effects of ivabradine and atenolol on HR should be compared over a 24 h period and include times of excitement and rest, such as during feeding and sleep.

A study in rats reported that prolongation of diastole by ivabradine increased sympathetic nerve activity by lowering diastolic blood pressure.^19^ Another previous study carried out in male rats with heart failure reported that administration of ivabradine suppressed the increase in sympathetic nerve activity.^20^ The reason why ivabradine changes autonomic nerve activity, even though it does not act on β‐receptors like atenolol, is that ivabradine may affect the intrinsic cardiac nerve system (ICNS).^21^ The ICNS has also been reported to be present in cats.^22^ In cats, chronic stimulation of the sympathetic nerve system may result in myocardial cell apoptosis and tissue fibrosis as well as increases in myocardial oxygen consumption.^22^ If the administration of ivabradine results in decreased sympathetic nerve activity in cats, it may be useful for the treatment of HCM cats.

In veterinary medicine, HR and HR variability (HRV) are used to estimate autonomic nervous system function.^23^ The HR is continually regulated by the action of the pacemaker while the sympathetic and vagal nerves maintain some degree of tonus.^24^ Increased sympathetic nerve activity results in an increased HR and the vagal nerve's inhibitory effect on the sympathetic nerve is predominant at rest. The HRV shows fluctuations in the cardiac cycle; HRV analysis is a non‐invasive test that quantifies autonomic nervous system function via two methods: time‐domain analysis and frequency‐domain analysis. The time‐domain analysis method uses variability in the RR interval on ECG resulting from changes in sympathetic and vagal nerve activity.^25^ Unlike the time‐domain analysis, the frequency‐domain analysis can quantify sympathetic and vagal nerve activity.^26^ The frequency band analysis is the preferred method to quantify HRV by using fast Fourier transformation. A 24 h Holter (24‐Holter) ECG is a non‐invasive test that features a small electrocardiography monitor that can record continuously for 24 h. The 24‐Holter ECG can evaluate HR and HRV for 24 h; the results can be interpreted as a quantitated autonomic nerve system function, for example sympathetic and vagal nerve activity.^27^

This study compared the impact of ivabradine and atenolol on HR and HRV over a 24 h period and during feeding and sleeping times via a 24‐Holter ECG in healthy cats. We hypothesized that ivabradine and atenolol would lower the HRs equally well, even at times of excitement and rest, such as during feeding and sleep. Furthermore, that ivabradine, unlike atenolol, would have an effect on HRV.

## MATERIALS AND METHODS

### Cats

In this study, with regard to animal welfare, a minimal number of cats that could be statistically analysed were included and then a crossover study design was adopted. Five healthy domestic short‐haired cats that were bred in our laboratory were used; with a mean age of 8.1 years (range 7.04−8.11 years) and mean weight at 4.3 kg (range 3.0−4.5 kg). These cats were regarded as clinically healthy based on a physical examination, complete blood count, biochemistry, echocardiography, electrocardiography and blood pressure measured using an oscillometric method. The cats were permitted unrestricted movement in individual stainless‐steel cages. Food and study medications were provided at 08:00 and 20:00; drinking water was provided ad libitum. After feeding and medicating the cats had no interaction with human beings. The study was compliant with the Guidelines for Institutional Laboratory Animal Care and Use of the Nippon Veterinary and Life Science University (Approval #2019S‐47).

### Study design

**FIGURE 1:**
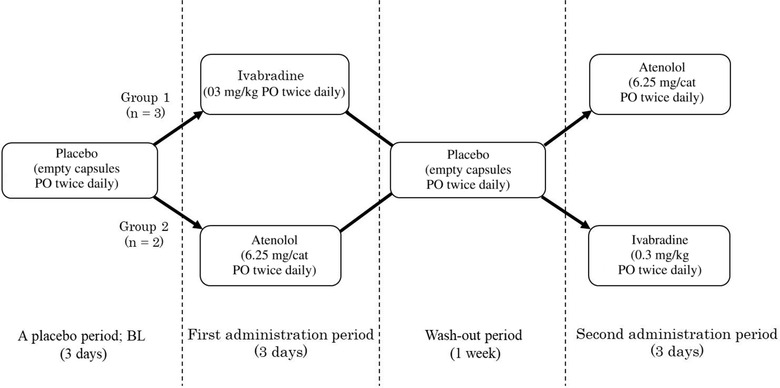
A summary of the study design. The baseline (BL) was the 24 h measurement period at the end of the 3 days of administration of a placebo (empy capsules per os (PO) twice daily)

The crossover was performed as follows: cats in group 1 were administered ivabradine (Procoralan; Servier, Suresnes, France; 0.3 mg/kg per os twice daily) followed by atenolol (Tenormin; AstraZeneca plc, Cambridge, UK; 6.25 mg/cat per os twice daily, range 1.3−2.0 mg/kg) and cats in group 2 were treated with ivabradine followed by atenolol (Figure 1).^14^, ^16^ The choice of which group each cat was placed in was randomly determined. Three cats were placed in group 1 and two cats in group 2. A placebo period was initiated 3 days before the start of the crossover test during which time empty capsules were given to cats every 12 h. Each drug was administered for 3 days.^28^, ^29^ The 3‐day trial period was separated by a 1‐week wash‐out period to prevent the first drug from affecting the duration of the next drug. All drugs were powdered and administered orally in dosage capsules (Matsuya, Osaka, Japan). The 24‐Holter ECG measurements were performed sequentially on each of the 3rd (last) day of the placebo, first and second drug administration periods (i.e. each cat wore a holter for three 24 h recording periods). For habituation, fitting with a holter jacket and placement of the 24‐Holter ECG device for cats were carried out during the placebo and administration of drug periods (3 days, respectively). A 24‐Holter ECG was also performed on the 7th day of the wash‐out period to check if the parameters had returned to their BL values during the wash‐out period.

### Evaluation parameters

Evaluation parameters included HR and HRV measurements (the time‐domain analysis included the standard deviation of all normal‐to‐normal intervals (SDNN), the square root of the mean squared differences between adjacent normal‐to‐normal intervals (RMSSD), the percentage of successive normal‐to‐normal interval differences that were greater than 50 ms (pNN50); the frequency‐domain analysis included the high‐frequency component (HF), the low‐frequency component (LF) and LF/HF) for 24 h, feeding and sleeping time periods. The HR and HRV measurements were obtained from the 24‐Holter ECG.

### 24‐Holter ECG

The 24‐Holter ECG was digital for the ECG registrations (QR2500; Fukuda M‐E kogyo, Tokyo, Japan). The sampling frequency was 150 Hz and the effective analogue/digital bit resolution was 0.5 mV. The device weighed 100 g and was carried by the cats in a garment with a pocket. Five electrodes were fixed to a shaved area that provided a two‐lead ECG directly visible on the laptop screen. No sedation drugs were used. The HR and HRV analyses were performed using an automatic analyser for the animals (HS1000; Fukuda M‐E kogyo).

Each parameter was compared separately for the whole 24 h, feeding and sleeping time periods. The data for the whole 24 h period were averaged using 30 min increments arbitrarily based on a resting state, low HR and RR interval variability over the whole 24 h period at different time points. The data for the feeding time were extracted as 30 min increments, for the periods at 08:00 and 20:00. The data for the sleeping time periods were extracted as 30 min increments, randomly selected and then averaged, at different time points examined from 01:00 to 04:00, when the room was completely dark and human traffic was restricted. The HF, LF and LF/HF were calculated by integrating the power spectral density in defined frequency bands (HF, 0.15–0.70 Hz; LF, 0.04–0.15 Hz).^27^, ^30^

### Statistical analysis

Statistical analyses were performed using SPSS Statistics software version 24.0 (IBM Corporation, Tokyo, Japan). The Kruskal–Wallis test was used to compare BL (end of the placebo period) and evaluation parameters (HR and HRV) for the whole 24 h period and for the feeding and sleeping time periods, with parameters recorded during each of the third (and last) 24 h period of the administration of both drugs. If a significant difference was observed the Steel–Dwass test was used to compare all pairs of medians. The same statistical analysis was used to compare differences for the BL/placebo period, in terms of HR and HRV, comparing the whole 24 h period with the feeding and sleeping periods.

A paired *t*‐test was used to compare the evaluation parameters between BL and day 7 of the wash‐out period to check if the parameters had returned to their BL values during the wash‐out period. Data are presented as median (minimum–maximum). A *p‐*value of <0.05 was considered statistically significant.

## RESULTS

**FIGURE 2:**
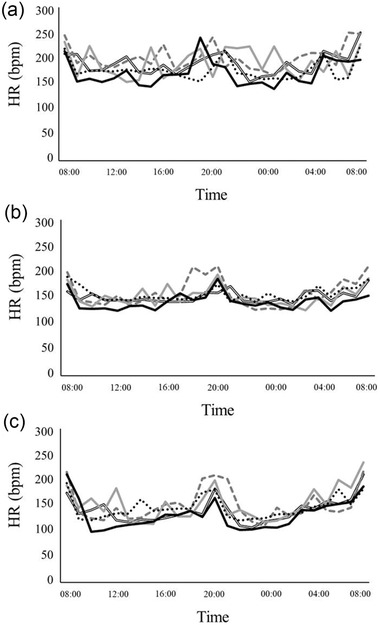
Mean hourly heart rate (beats per minute, bpm) of each cat (*n* = 5) at baseline (a) and after administration of 3 days of receiving ivabradine (b) and atenolol (c)

The mean hourly HR (bpm) of each cat (*n* = 5) at BL and after the administration of 3 days of ivabradine and atenolol, is shown in Figure 2a–c. The HR tended to increase at feeding times (8:00, 20:00) when all of the cats were observed purring and moving around the cage.

When reviewing only the data for the BL time point, the HR and LF/HF were significantly higher, while the SDNN, RMSSD, pNN50 and HF were significantly lower, during the feeding period, compared with the overall 24 h period and the sleeping period (Table 1, *p* < 0.05).

**TABLE 1:** The heart rate (HR) and heart rate variability (HRV) measurements over 24 h period as the baseline (end of the 3‐day placebo period)

	24 h	Feeding time	Sleeping time
HR (beats per minute)	187.18 (171.79–193.19)	216.43^a^ (207.23–223.04)	182.98^b^ (161.31–185.15)
Standard deviation of all normal‐to‐normal intervals (SDNN) (ms)	25.83 (23.64–28.74)	13.53^a^ (12.49–18.17)	29.12^b^ (27.16–35.32)
Square root of the mean squared differences between adjacent normal‐to‐normal intervals (RMSSD) (ms)	20.33 (16.31–23.24)	10.60^a^ (7.98–11.21)	23.0^b^ (19.17–27.95)
The percent of successive normal‐to‐normal interval differences that were greater than 50 ms (pNN50) (%)	1.57 (0.99–4.56)	0.08^a^ (0–0.30)	1.59^b^ (0.94–5.63)
High‐frequency component (HF) (ms^2^)	482.46 (249.30–631.93)	73.44^a^ (62.74–93.59)	159.33^b^ (129.32–231.57)
Low‐frequency component (LF) (ms^2^)	147.77 (99.22–176.87)	28.80^a^ (24.38–44.83)	25.99^a^ (23.58–55.24)
LF/HF	0.33 (0.24–0.60)	1.66^a^ (1.24–1.97)	0.24^b^ (0.22–0.33)

*Note*: Data are represented as the median (minimum–maximum).

^a^ *p* < 0.05 compared with the whole 24 h period.

^b^ *p* < 0.05 compared with feeding time period.

The HRs over 24 h and during the feeding and sleeping times, recorded after the administration of 3 days of ivabradine and atenolol, were significantly lower than the BL values. The degree to which HR was reduced from BL were comparable for both ivabradine and atenolol (Tables 2, 3, 4).

**TABLE 2:** Heart rate (HR) and heart rate variability (HRV) measurements, over a 24 h period, comparing parameters with baseline, for cats treated with ivabradine and atenolol

	Baseline	After the administration of ivabradine	After the administration of atenolol
Heart rate (HR) (beats per minute)	187.18 (171.79–193.19)	143.14^a^ (134.44–149.02)	151.44^a^ (144.96–153.97)
Standard deviation of all normal‐to‐normal intervals (SDNN) (ms)	25.83 (23.64–28.74)	35.41^a^ (32.89–42.04)	26.19^b^ (24.56–31.45)
Square root of the mean squared differences between adjacent normal‐to‐normal intervals (RMSSD) (ms)	20.33 (16.31–23.24)	34.84^a^ (30.31–41.56)	24.53^b^ (21.77–29.56)
The percent of successive normal‐to‐normal interval differences that were greater than 50 ms (pNN50) (%)	1.57 (0.99–4.56)	13.56^a^ (6.45–16.20)	4.23^b^ (1.02–6.37)
High‐frequency component (HF) (ms^2^)	482.46 (249.30–631.93)	950.77 (387.39–1536.80)	483.07 (404.34–771.89)
Low‐frequency component (LF) (ms^2^)	147.77 (99.22–176.87)	291.27^a^ (202.25–293.48)	162.26^b^ (62.33–208.53)
LF/HF	0.33 (0.24–0.60)	0.30 (0.18–0.52)	0.27 (0.15–0.46)

*Note*: Data are represented as the median (minimum–maximum).

^a^ *p* < 0.05, compared with baseline (end of a 3‐day period of receiving a placebo).

^b^ *p* < 0.05, compared with after the administration of ivabradine.

**TABLE 3:** The heart rate (HR) and heart rate variability (HRV) measurements, recorded during feeding times, comparing parameters with baseline, for cats treated with ivabradine and atenolol

	Baseline	After administration of ivabradine	After administration of atenolol
Heart rate (HR) (beats per minute)	216.43 (207.23–223.04)	176.70^a^ (142.26–210.87)	174.29^a^ (153.16–188.89)
Standard deviation of all normal‐to‐normal intervals (SDNN) (ms)	13.53 (12.49–18.17)	17.68 (15.05–65.66)	13.02^b^ (9.22–21.81)
Square root of the mean squared differences between adjacent normal‐to‐normal intervals (RMSSD) (ms)	10.60 (7.98–11.21)	15.53 (11.56–24.07)	10.37 (6.87–39.52)
The percent of successive normal‐to‐normal interval differences that were greater than 50 ms (pNN50) (%)	0.08 (0–0.30)	0.81 (0.09–6.05)	0.22 (0.01–9.78)
High‐frequency component (HF) (ms^2^)	73.44 (62.74–93.59)	118.23 (51.84–327.76)	39.50^b^ (17.50–573.63)
Low‐frequency component (LF) (ms^2^)	28.80 (24.38–44.83)	118.02 (61.32–213.56)	71.23 (32.13–119.04)
LF/HF	1.66 (1.24–1.97)	0.64 (0.32–2.51)	1.53 (0.19–3.39)

*Note*: Data are represented as the median (minimum–maximum).

^a^ *p* < 0.05, compared with baseline (end of a 3‐day period of receiving a placebo).

^b^ *p* < 0.05, compared with after the administration of ivabradine.

**TABLE 4:** The heart rate (HR) and heart rate variability (HRV) measurements, recorded during sleeping periods, comparing parameters with baseline, for cats treated with ivabradine and atenolol

	Baseline	After administration of ivabradine	After administration of atenolol
Heart rate (HR) (beats per minute)	182.98 (161.31–185.15)	136.44^a^ (111.76–144.70)	144.40^a^ (130.61–149.21)
Standard deviation of all normal‐to‐normal intervals (SDNN) (ms)	29.12 (27.16–35.32)	48.47^a^ (40.12–70.30)	27.41^b^ (24.93–35.02)
Square root of the mean squared differences between adjacent normal‐to‐normal intervals (RMSSD) (ms)	23.0 (19.17–27.95)	40.97^a^ (34.94–86.68)	27.90^b^ (22.71–48.85)
The percent of successive normal‐to‐normal interval differences that were greater than 50 ms (pNN50) (%)	1.59 (0.94–5.63)	17.64^a^ (5.37–37.92)	4.49^b^ (0.19–11.18)
High‐frequency component (HF) (ms^2^)	159.33 (129.32–231.57)	1671.20^a^ (961.84–2566.60)	428.46^b^ (143.67–742.68)
Low‐frequency component (LF) (ms^2^)	25.99 (23.58–55.24)	350.87^a^ (306.86–668.55)	126.46^b^ (62.70–213.99)
LF/HF	0.24 (0.22–0.33)	0.23 (0.18–0.46)	0.31 (0.15–0.48)

*Note*: Data are represented as the median (minimum–maximum).

^a^ *p* < 0.05, compared with baseline (end of a 3‐day period of receiving a placebo).

^b^ *p* < 0.05, compared with after the administration of ivabradine.

For the 24 h measurement period with administration of ivabradine, the HRV, in terms of SDNN, RMSSD, pNN50 and LF, were significantly elevated compared to BL values and those recorded during the administration of atenolol (Table 2, *p* < 0.05).

The HRV for feeding time for ivabradine was significantly increased for SDNN and HF, compared to atenolol (Table 3, *p* < 0.05).

The HRV for sleeping time after the administration of ivabradine, in terms of SDNN, RMSSD, pNN50, HF and LF, were significantly higher than the BL values and those recorded during the administration of atenolol (Table 4, *p* < 0.05).

There was no significant difference in HR or HRV between the BL period and the end of the wash‐out period. It was, therefore, presumed that the effect of the drug administered in the first administration period had disappeared during the wash‐out period (*p* < 0.05).

## DISCUSSION

As hypothesized, the present study showed that ivabradine and atenolol decreased HR during both feeding and sleeping times. The HRV was also shown to be altered by ivabradine, as hypothesized. Additionally, the study showed that the effect was greater during the sleeping than the feeding time. To the best of the authors’ knowledge, this is the first study that has investigated the effect of ivabradine on HRV in cats.

Comparison of 24 h, feeding time and sleeping time during BL showed that HR and LF/HF were significantly higher, while SDNN, RMSSD, pNN50, HF and LF were significantly lower for the feeding time, than the whole 24 h period and during the sleeping time. The behaviour of the cats at feeding time was indicative of an excited state. SDNN and RMSSD are used as indicators of the degree of HRV, while pNN50 indicates vagal nerve activity.^23^ The HF parameter is used as an indicator of vagal nerve activity and LF is used to indicate sympathetic and vagal nerve activity.^31^, ^32^ Thus, these indicate an increase in sympathetic nerve activity and/or a decrease in vagal nerve activity. The significant decrease in the LF/HF may reflect an increase in sympathetic nerves activity and a decrease in vagal nerves activity during the feeding time compared with BL. The changes in HR and HRV during feeding time observed in the present study are similar to those reported in previous studies comparing HR and HRV in cats at home and in hospital, where the cats were excited in the clinical hospital setting.^27^ Thus, the results of the present study indicate that the cats were in an excited state during feeding time. Moreover, the results are similar to a study that reported the population of cats showed a peak of activity especially before sunrise and during food renewal.^18^

In cats, excitement has been reported to increase sympathetic nerve activity which then increases HR. In this study, the HRs during the feeding time, during the periods of administration of ivabradine and atenolol, showed a significantly lower value compared with the BL. A study investigating the effect on HR reduction by ivabradine after an acoustic startle reduction only evaluated instantaneous HR rather than HR over time.^16^ Therefore, it should be investigated whether the HR‐reducing effect of ivabradine and atenolol is also observed under conditions of increased sympathetic activity. Results of this study revealed that ivabradine and atenolol medication was associated with reduced HR, compared with BL, even under conditions when there was increased sympathetic nerve activity. In a study of ivabradine and atenolol in healthy cats under anaesthesia, only ivabradine was able to reduce dobutamine‐induced tachycardia.^15^ Possible reasons for the discrepancy between our results and those of previous studies include the effects of anaesthesia on cardiac function and possible interactions with the effects of the drugs, which were not clarified in the previous study.

Since the degree of HR‐reducing action was the same despite the different mechanisms of action even when sympathetic nerve activity is increased by excitement, it seems that either of the two drugs can be administered with the expectation of a reduction in HR. However, atenolol is an inhibitor of β‐receptor signalling; inhibition of the β1‐receptor may result in a decrease in myocardial contractility.^33^ As such, atenolol should not be administered to cats with reduced myocardial contractility or congestive heart failure.^1^, ^4^ By contrast, ivabradine has no impact on myocardial contractility or the vasculature as it acts on *I*_f_ channels which are abundant in the sinus node; ivabradine treatment serves to raise the cell membrane potential, thereby suppressing activity at the sinus node.^12^ Consequently, ivabradine should be useful for the treatment of tachycardia regardless of the presence or absence of congestive heart failure. This suggests that ivabradine may be more appropriate than atenolol in cats with congestive heart failure.

The HR has been reported to tend to decrease during sleep in cats.^17^ Bradycardia is an adverse event of ivabradine and atenolol in human beings.^12^, ^34^ Accordingly, it was anticipated that cats may show bradycardia with ivabradine or atenolol during sleep when HR was decreasing. However, in the cats of this study, the degree of HR‐reducing effect of ivabradine and atenolol did not change and was not associated with a tendency to bradycardia. From these results, ivabradine and atenolol have been shown to reduce the HR of cats to a certain level even during periods of excitement and do not cause bradycardia even during sleep.

In this study, ivabradine altered HRV, whereas atenolol did not alter HRV. The HF reflects not only an increase in vagal nerve activity but also changes in blood pressure in the cat.^30^ Previous studies in rats have shown that treatment with ivabradine decreased only diastolic blood pressure measured spectroscopically, with concomitant activation of the sympathetic nervous system.^19^ In cats, it has been reported that ivabradine did not alter blood pressure, however, the diastolic blood pressure was unknown because measurement requires an invasive procedure.^14^ Therefore, the HF in the present study may have been related to blood pressure changes. However, SDNN, RMSSD, pNN50 and LF, which are used to quantify vagal activity, were also elevated, suggesting that HF was elevated due to the increased vagal activity rather than blood pressure variation.^27^, ^31^ In the present study, HRV was also decreased during excitement, which may have been due to increased sympathetic activity in the cats, as mentioned earlier. The HRV parameters for the BL and after ivabradine and atenolol therapy, were not significantly different at feeding time, whereas the most significant differences were observed at the sleeping time when the sympathetic activity tended to decrease. This suggests that the changes in HRV parameters after ivabradine administration in the present study may be due to a decrease in sympathetic activity and/or an increase in vagal activity.

Our results suggest that treatment with ivabradine decreases the activity of the sympathetic nerve system and/or increases activity of the vagal nerve system, similar to a previous study in human subjects.^35^ In human beings, ICNS is involved as a mechanism in which ivabradine acts on the autonomic nerves despite the *I*_f_ channel blocker.^21^ In human beings, ivabradine reduces HR and prolongs diastole by blocking the *I*_f_ channel distributed in the sinus node, resulting in increased coronary perfusion; reduced release of ATP degradation products and lactate production;^36^ increased end‐diastolic volume, wall stress and stroke in patients with myocardial ischaemia.^37^ Information on such changes in the heart caused by ivabradine is sent from the ICNS to the brain via the vagal nerve. Suppression of autonomic nerve function based on the information sent by ICNS to the brain has been believed to result in decreased sympathetic nerve activity and increased vagal nerve activity.^21^ Although we included healthy cats in this study, it has been reported that even in healthy human beings, a decrease in HR causes the enlargement of the end‐diastolic volume with increased wall tension, which adjusted stroke volume.^38^ Therefore, the above mechanism may be related to the results of this study. Furthermore, ivabradine administered to healthy cats may have an impact on autonomic nerve function and may be beneficial in suppressing the progression of heart disease. Further research is needed on the effects of ivabradine administration on HRV in cats.

There were several limitations to this study including that a small number of cats were included. Thus, we may have been unable to identify the presence of statistical significance. However, this study identified significant differences in some evaluation parameters even with the small number of cats. With careful consideration of animal welfare, the number of cats included was deemed appropriate. Additionally, as we used clinically healthy cats in this study, it is difficult to extrapolate these results to cats with cardiomyopathy. We did not use clinical cases in this study because there is a difference in the degree of autonomic nerve system activity and likely substantial differences in their home environment/husbandry. The autonomic nerve system is sensitive to the environment.^39^ Since this study aimed to compare the autonomic nerve system when administering ivabradine and atenolol, the husbandry condition, light/dark cycle and feeding time should be controlled. Thus, experimental cats managed under the same facility with the unified light/dark cycle and feeding time were used. The effect of ivabradine on HRV in cats with cardiomyopathy should be investigated in the future based on the results of this study.

The possible impact of human interaction with the cats was blocked except for the feeding time, so that cat behaviour was not monitored for the whole of the 24 h period of the present study. It was not clear whether all cats were always asleep during the proposed sleeping time. Therefore, there may not have been a significant difference in HR and HRV between 24 h and sleeping time during the BL assessment period. A different result might have been obtained if the data for sleeping were collected when the cats were definitely asleep.

The results from this study suggest that ivabradine and atenolol significantly reduced HR regardless of excitement and rest, such as feeding and sleeping times; their effects were comparable in healthy cats. Only ivabradine led to an increase in HRV, the effect was particularly notable at rest.

## FUNDING INFORMATION

This research did not receive any specific grant from funding agencies in the public, commercial or not‐for‐profit sectors.

## CONFLICTS OF INTEREST

The authors declare they have no conflicts of interest.

## ETHICS STATEMENT

Not applicable.

## Data Availability

The data that support the findings of this study are available from the corresponding author, Ogawa M, upon reasonable request.
